# COVID-19 response in WHO African Region: country and regional office experiences

**DOI:** 10.11604/pamj.supp.2022.41.2.34497

**Published:** 2022-03-29

**Authors:** Abdou Salam Gueye

**Affiliations:** 1 WHO African Regional Office, Brazzaville, Congo

**Keywords:** COVID-19, SARS-CoV-2, ACT-Accelerator, WHO African Region, pandemic preparedness, pandemic response

## Editorial


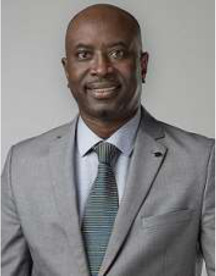


The African region faced a large-scale public health crisis, with the outbreak of coronavirus disease 2019 (COVID-19). As of February 16, 2022, the region recorded 7,944,049 confirmed cases with case fatality of 2.1 percent [1]. The outbreak of COVID-19 in Africa created a sense of urgency, and inspired calls for a coordinated regional response to stop the pandemic and avert the catastrophic predictions and modelling churned out based on the relatively weak health systems [2-4].

The WHO African region adopted and implemented the new WHO ACT-Accelerator strategy in its response to the pandemic [5]. New ACT-Accelerator strategic plan set out urgent actions to address crucial gaps in access to COVID-19 tests, treatments, vaccines, and personal protective equipment in low- and middle-income countries, using the latest epidemiological, supply and market information, among other actions. These actions were aimed at addressing inequitable access to COVID-19 tests, treatments and vaccines and reduce the risk of prolonging the pandemic in the region as well as avert the risk of emergence of new, more dangerous variants that could evade current tools to fight the disease. Countries in the African region responded to the threat of the pandemic by mobilizing resources at their disposal to avert the predicted negative impacts.

The countries mounted and implemented several actions in response to the outbreak. Some actions entailed the use of innovative strategies in addressing transmissibility and spread of the virus, reaching the populations with vaccines, diagnostics, and therapeutics. Others were on surveillance and testing, infection prevention and control, public health and social measures as well as contact tracing, among others. The Incident Management Support Team (IMST) of the WHO AFRO further enabled the countries with evidence-driven innovations and guidelines in responding to the COVID-19 pandemic.

Several experiences were gathered that could be useful for future public health interventions. This special issue of the Pan African Medical Journal documents the of the key lessons and experiences gathered from response to the COVID-19 pandemic in countries as well as the WHO AFRO IMST. The papers, written by those who were actively engaged in response to epidemics in countries, cover critical topics from preparedness and response to disease outbreaks, strengthening surveillance systems and integration as well as strengthening health workforce against pandemics. The comprehensive analysis of the success stories made in pandemic response will no doubt enhance response to future public health events within and outside the region. These articles therefore constitute a case of turning tragedy to gains!
